# Visualizing software refactoring using radar charts

**DOI:** 10.1038/s41598-023-44281-6

**Published:** 2023-11-09

**Authors:** Abdel-Rahman Al-Ghuwairi, Dimah Al-Fraihat, Yousef Sharrab, Huda Alrashidi, Nouf Almujally, Ahmed Kittaneh, Ahmed Ali

**Affiliations:** 1 Department of Software Engineering, Faculty of Prince Al-Hussien Bin Abdallah II for Information Technology, The Hashemite University, Zarqa, Jordan; 2 Department of Software Engineering, Faculty of Information Technology, Isra University, Amman, Jordan; 3 Department of Data Science and Artificial Intelligence, Faculty of Information Technology, Isra University, Amman, Jordan; 4 Faculty of Information Technology and Computing, Arab Open University, Ardiya, Kuwait; 5 Department of Information Systems, College of Computer and Information Sciences, Princess Nourah Bint Abdulrahman University, P.O. Box 84428, Riyadh, 11671 Saudi Arabia

**Keywords:** Software, Computer science

## Abstract

Refactoring tools have advanced greatly and are being used in many large projects. As a result, a great deal of information is now available about past refactoring and its effects on the source code. However, when multiple refactoring is performed at once, it becomes more difficult to analyze their impact. Refactoring visualization can help developers create more maintainable code that is easier to understand and modify over time. Although there is an increasing interest in visualizing code changes in software engineering research, there has been relatively little research on visualizing the process of refactoring. In this paper, we propose a Radar Chart Refactoring Visualization (RcRV) approach to visualize software refactoring of source code across multiple software releases. Radar charts are a form of 2D visualization that can show multiple variables on a single chart. The RcRv receives input from developers or through refactoring identification tools, such as Ref-Finder, to generate charts. The generated charts can show the changes made during the refactoring process, highlighting areas of the trend of refactoring over evolution for multiple refactoring, multiple methods, and multiple classes. The evaluation study conducted to assess the usefulness of the RcRV tool has shown that the proposed tool is useful to developers, appealing, and easy to use. The proposed method of visualization can be beneficial for developers and maintainers to detect design violations and potential bugs in the code, thus saving time and effort during the development and maintenance process. Therefore, this research presents a significant contribution to the software engineering field by providing developers with an efficient tool to enhance code quality and maintainability.

## Introduction

Software refactoring is a well-known practice that is used to improve the quality of software systems. Refactoring involves making changes to the code structure without affecting the system's functionality^1^. It is essential for software engineers to refactor their code to ensure that it remains maintainable, scalable, and reusable^2,3^. Recently, contemporary refactoring tools have been advancing rapidly and are now being widely utilized in several large projects. Consequently, a vast amount of information is available on past refactoring activities and their effects on the source code^4^. However, when multiple refactoring is performed simultaneously, it becomes increasingly challenging to analyze their impact. Researchers propose the use of visualization tools for refactoring to improve code comprehension and make it easier for developers to navigate complex codebases^5^. Visualizations are essential tools for software engineers to understand the impact of their code changes. Visualizing the refactoring can be especially helpful for new developers who are unfamiliar with the codebase^6^.

Refactoring visualization is vital for developers because it allows them to understand the structure of the code. Additionally, visualization can aid developers in detecting potential code issues that violate design principles, pinpointing code paths that are vulnerable to bugs, and ultimately reducing the time required during the development process^6^. Despite the increasing interest in visualizing code modifications in software engineering research, there has been limited research on visualizing the refactoring process. Further, several software tools have been developed to support refactoring but there is a lack of effective visualizations to aid developers in understanding the impact of these changes^7^.

In this paper, we propose a novel approach to visualize software refactoring using radar charts. A radar chart, also known as a spider chart or a web chart, can be used to visualize the impact of refactoring on multiple dimensions of the codebase simultaneously^8^. This type of chart can be useful when developers want to compare the relative strengths and weaknesses of different aspects of the code before and after refactoring. For example, a radar chart could be used to compare the complexity, maintainability, test coverage, and code smells of the code before and after refactoring. Further, the chart would show each of these dimensions as a separate axis on the chart, with a line connecting the data points for the before and after refactoring states. This would allow showing immediately which aspects of the code have improved the most and which areas may still need additional attention.

Our approach, called Radar Chart Refactoring Visualization (RcRV), visualizes the refactoring of source code across multiple releases. The approach presents refactoring as rings, which allows for easy tracking of the trend of refactoring across multiple methods and classes. The graphs produced offer a condensed view of the refactoring progression, facilitating an examination of prior actions and forecasting future directions. The approach can be used to analyze four cases of past behavior related to multiple refactoring, methods, classes, and releases as follows:

- Case 1: visualization of multiple refactoring for multiple methods in one specific class with a refactoring counter.
- Case 2: visualization of one refactoring type for multiple methods in multiple classes with a refactoring counter.
- Case 3: visualization of multiple refactoring for multiple classes with a refactoring counter.
- Case 4: Visualization of multiple refactoring over evolution with a refactoring counter.

The proposed visualization approach for refactoring using radar charts can be a good choice for refactoring projects where there are multiple dimensions of the code that need to be improved, and where it's important to see how the improvements in each dimension relate to each other. The motivation behind this research stems from the desire to increase the utilization of automated refactoring tools and improve developers' proficiency in automated refactoring practices. The proposed tool aims to empower developers by providing them with a visualization approach that provides a clear and comprehensive understanding of the refactoring process, enabling them to make informed decisions, and optimize their codebase effectively. Radar charts are a visually appealing and straightforward method that has not been explored in this context before. To the best of our knowledge, there has been no previous research that visualizes software refactoring using radar charts. Additionally, the proposed tool has been empirically evaluated with developers to evaluate the usefulness of this visualization tool and is considered useful to developers, appealing, and easy to use.

The rest of the paper is organized as follows: Section “Background” provides the research background. Section “Related work” reviews the literature and related work. Section “Research methods” presents research methods. Section “RcRV approach and visualization results” introduces the proposed approach and discusses generated graphs. Finally, Section “Evaluation of the refactoring visualization tool” concludes the study, and Section “Implications” outlines limitations and future work.

## Background

Refactoring is the process of improving the quality of existing code by making modifications to its structure, design, and implementation without altering its external behavior or functionality^1^. The primary goal of refactoring is to improve the code's readability, maintainability, and extensibility while reducing its complexity and eliminating code smells.

In software engineering, code refactoring is an essential technique used to optimize and maintain software systems. By continuously improving the code quality, refactoring reduces technical debt and helps developers to add new features and fix bugs more efficiently. Additionally, refactoring can also lead to better software design, better performance, and increased code reuse^9^.

Some common refactoring techniques include simplifying code, removing duplication, improving naming conventions, and applying design patterns^10^. Refactoring is typically done in small, incremental steps, and each change is tested to ensure that the code's behavior remains unchanged. Refactoring is often an ongoing process that occurs throughout the software development lifecycle, as code evolves and requirements change^11^.

Observing the progress of large software systems, particularly object-oriented projects, presents a challenge due to their vastness, further complicated by the increased amount of data to be analyzed for every release being examined. Software metrics and visualization are two useful techniques used to track refactoring^12^. Software metrics, such as complexity metrics, provide condensed information on source code data, but their huge tables can be challenging to interpret, and new metrics are often of questionable usability and fuzzy definition^13^. Software refactoring visualization is a technique used to visualize the structure of code during the refactoring process. The goal of visualization is to provide a better understanding of the codebase and to help identify areas that need improvement. By visualizing the code, developers can more easily see how different pieces of code are connected, and how changes in one area can affect the rest of the system^14^.

There are many tools available for visualizing software refactoring. Some tools generate diagrams that show the relationships between classes, methods, and other code elements^15^. These diagrams can help developers understand the overall structure of the codebase and identify areas that may be overly complex or tightly coupled. Other tools provide real-time feedback on the effects of code changes. For example, as a developer makes changes to the codebase, the tool may highlight areas that are impacted by those changes^16^. This can help developers avoid introducing new bugs or other issues during the refactoring process^17^.

Visualization tools can also help with code comprehension, as they provide a more intuitive way to navigate and understand complex codebases. This can be especially helpful for new team members who are not yet familiar with the codebase^9^. Overall, software refactoring visualization is a powerful technique that can help developers improve the quality of their codebases and reduce technical debt. By providing a better understanding of the code, developers can make more informed decisions about how to refactor and improve their software.

In software refactoring, polymetric visualization has been implemented, whereby nodes correspond to modules with the width determined by the number of classes they contain, the height by the number of files they contain, and the color by the number of directories they contain^18^. However, when dealing with extensive graphs and numerous releases, identifying disparities and patterns in the metrics of nodes and arcs becomes more intricate, if not unfeasible. Radar chart is a more efficient visualization technique that can be used in software refactoring to help identify and improve the design of software systems^8^. A radar chart, also known as a spider chart or a star chart, is a graphical representation of data that shows multiple variables plotted on a circular grid with each variable represented by an axis that emanates from the center of the chart^19^. Radar chart refactoring is used to analyze the design of a software system by plotting different software metrics on the axes of a radar chart. The metrics can include code complexity, code coupling, test coverage, and other quality metrics^20^.

The radar chart is used to identify areas of the system that may need refactoring or improvement. For example, if the chart shows that a particular module of the software has high code complexity, low test coverage, and high coupling to other modules, this may indicate that the module needs to be refactored to improve its maintainability and reliability. Radar chart refactoring can be a useful tool for software engineers to gain insight into the design of complex software systems and identify areas for improvement^21^.

## Related work

Maintaining good design is crucial in software development. As previously noted, refactoring is a technique that enables developers to enhance the design of their software systems without altering their behavior during the development process. Refactoring enhances the system's higher-level view through the implementation of design-oriented enhancements, including the reorganization and restructuring of fields, methods, and classes^1^. This process also improves code readability and the potential for modifications. The motivation behind such modifications lies in the enhancement of static metrics and quality aspects like coupling, cohesion, and complexity, as well as in the elimination of code smells^22,23^.

Refactoring can be executed either manually or through automation. As software systems expand, the process of manual refactoring escalates in complexity. Manual refactoring can be time-consuming and challenging for developers who lack experience or familiarity with the codebase. Moreover, its subjective nature and the potential for errors further escalate these challenges^7^. As a result, automated refactoring has received substantial attention to address the intricacies posed by growing software complexity. In response, research have been dedicated to automating the refactoring process, leading to the development of various tools and frameworks. These resources are specifically designed to provide developers with automated assistance and recommendations for executing refactoring operations, thereby mitigating the complexities associated with evolving software systems. JDeodorant, TrueRefactor, and Eclipse Refactoring are examples of popular tools that have been utilized by software engineering community^7^. These tools can automatically identify design antipatterns and provide a diverse range of potential corrective refactoring. Subsequently, developers select the most suitable refactoring tasks that align with their design preferences.

Automated approaches can help identify opportunities for refactoring and hold the potential to enhance refactoring’s efficiency significantly and accurately^24^. Despite these advantages, developers have exhibited lower adoption rates of refactoring tools than anticipated^6^. Developers express concerns regarding the extensive alterations these tools introduce to the current design. This hesitance arises from the fact that while developers seek to optimize their codebase, they also desire to retain familiarity with their existing design. Consequently, researchers encounter a challenging task: enhancing the accuracy and automation of refactoring while concurrently improving the adoption of automation tools.

To address the issue of low adoption of automated refactoring tools, researchers suggest the use of visualization tools for software refactoring which can aid in code comprehension by offering a more natural and intuitive way to navigate and comprehend complex codebases^6,25,26^. This can be particularly useful for new developers who are not yet acquainted with the codebase. Despite the growing interest in visualizing code modifications in software engineering research, there has been comparatively little research on visualizing the act of refactoring. An overview of related work in the field of visualization of software refactoring, along with a brief discussion of the limitations of existing visualization techniques, is presented as follows.

Pinzger et al.'s^27^ research introduces the RelVis visualization method, which aims to create succinct and comprehensive graphical representations of source code and release history data for up to n releases. The approach employs Kiviat diagrams to showcase metrics for source code elements and their interconnections as annual rings. The diagrams emphasize instances of positive and negative performance for each element, facilitating the identification of critical trends in both entities and relationships. This knowledge can prove useful in identifying areas of the code that necessitate refactoring before further development of the system. The paper also includes contextual information and an assessment of the method's effectiveness, utilizing a substantial open-source software project.

Identifying the types of refactoring that occurred between two program versions was studied by Kim et al.^28^. They introduced an Eclipse plug-in called Ref-Finder, which uses a template-based approach to identify complex refactoring between two program versions. It represents each refactoring type using template logic rules. Ref-Finder utilizes a logic programming engine to infer specific refactoring instances. Further, the tool supports 63 types of refactoring in comparison to other tools providing the most comprehensive coverage.

Another study conducted by^29^ presents a framework for a visualization approach that helps software maintainers locate and comprehend bad smells in code, which can then be eliminated through refactoring. The visualization method involves displaying object-oriented code elements and highlighting any existing bad smells. Specifically, the proposed visualization depicts classes as buildings, and bad smells are represented by letter avatars based on their initials. These avatars are then shown as warning signs on the corresponding buildings. The results of their study indicate that these visualizations can reduce the time required for maintainers to comprehend bad smells.

The study of Rodriguez et al.^30^ introduced a web-based tool called VizSOC to help software developers identify refactoring opportunities in service-oriented applications. The tool takes “Web Service Description Language” (WSDL) documents as input, detects anti-patterns, and suggests ways to resolve them, providing a list of refactoring suggestions to start the refactoring process. The Hierarchical Edge Bundles (HEB) visualization technique was used and was evaluated using two real-life case studies, measuring the number of anti-patterns detected and the performance of clustering algorithms based on internal validity criteria. The findings indicate that VizSOC is a useful tool for detecting refactoring opportunities, enabling developers to reduce the effort required in the detection process.

The research presented by Cassell et al.^31^ used clustering techniques to solve the challenges faced by developers when trying to reallocate members of large and complex object-oriented classes. The authors introduced the ExtC Visualizer as a tool to help programmers understand the class structure, emphasizing critical features of the members and their interrelationships, and visualizing how clustering algorithms group the members. The proposed tool can aid developers in selecting the most appropriate techniques for refactoring large classes. Further, Bogart et al.^6^ claimed that developers may not trust automated refactoring tools and proposed a visualization approach to help developers understand suggested operations and increase familiarity with automated refactoring tools. The approach is manually validated, and options for further experimentation were identified.

Visualizing software refactoring is a complex task that involves representing code changes in a way that is easy for developers to understand and use. Overall, while there has been progress in visualizing software refactoring, there are still many challenges and limitations that need to be addressed to make these visualizations more useful for developers in practice^7^. The challenges and limitations of existing research in visualizing software refactoring include the difficulty in interpreting complex refactoring: some refactoring is more complex than others, and it can be difficult to represent them visually in a way that is easy for developers to understand^32^. This can limit the usefulness of visualizations for more complex refactoring tasks. The lack of standardization, complexity of code, limited scope of existing techniques, lack of integration with development tools, and difficulty in evaluating effectiveness are all challenges that need to be addressed to make visualizations of software refactoring more useful for developers in practice^33^.

The primary goal of this research is to support developers in becoming more comfortable, confident, interactive, and familiar with automated refactoring. Our visualization approach would extend and complement the functionalities of existing automated visualization tools through visualizing the refactoring evolution in the software system, facilitating analysis of historical trends and prediction of future directions, and identifying potential refactoring candidates before further system evolution. Further, the existing study contributes to the software engineering field by proposing a visualization approach that introduces the possibility of visualizing multiple refactoring operations, in contrast to the conventional practice that attempts to sequentially visualize refactoring without a comprehensive plan. The proposed approach aims to minimize the human effort required when dealing with automated refactoring tools and optimize the software system. Moreover, to the best of our knowledge, no previous research has employed radar charts to visualize software refactoring. Radar charts are a visually appealing and straightforward method that has not been explored in this context before. In the next section, our proposed Radar Chart Refactoring Visualization (RcRV) approach is discussed.

## Research methods

To construct our RcRV visualization tool, we procured the Refactoring data, which serves as the input for the tool. The Refactoring data was sourced from the Evolutionary data, which encompasses all modifications made to the software system over a period of time. The Refactoring data was obtained from the Evolutionary data using Refactoring identification tools, such as Ref-Finder. Subsequently, we utilized the Refactoring data as the input for the RcRV approach and to generate visualizations.

**Figure 1:**
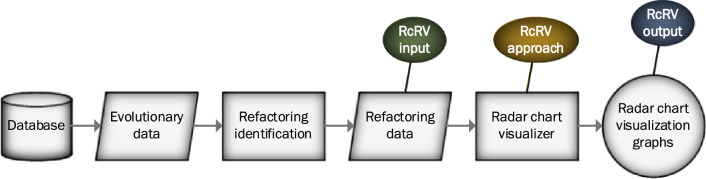
Research steps.

The radar chart visualizer from the plotly library in Python was employed to generate visualizations of the Refactoring data. Details on the implementation of this process are presented in the subsequent subsection. The output of the RcRV approach is visualized data, which comprises graphs that display Refactoring information for two or more software releases utilizing radar charts. The sequential steps we followed in our research are illustrated in Fig. 1. Additionally, a description of the dataset utilized in this research is provided.

As outlined in Fig. 1, the sequential steps taken to analyze data and generate radar chart visualizations using the Refactoring-centric Radar Visualization (RcRV) approach can be summarized as follows:

- STEP 1. Data Collection and Database Setup: The research process begins with the collection of relevant data, which is stored in a designated database. This data encompasses information crucial for the analysis of software evolution and refactoring activities.
- STEP 2. Analysis of Evolutionary Data: The collected data undergoes a comprehensive analysis to discern patterns and trends in software evolution. This step involves examining changes in the codebase over time, identifying key points of evolution, and gaining insights into the development process.
- STEP 3. Refactoring Identification: To identify instances of refactoring within the codebase, specialized tools such as Ref-Finder are employed. These tools help in the systematic detection and classification of refactoring activities carried out during the software development lifecycle.
- STEP 4. Refactoring Data Collection (RcRV Input): The refactoring data, obtained from the previous step, serves as the input for the RcRV tool. This data constitutes the foundation upon which the RcRV approach will operate.
- STEP 5. Application of RcRV Approach: Building upon the refactoring data, this step applies the RcRV to process and analyze the data. The RcRV approach is discussed in detail in the following section.
- STEP 6. Generation of Radar Chart Visualizations (RcRV Output): The results of the RcRV approach appear in the form of radar chart visualizations.

### Dataset description

The dataset utilized in this was adopted from a previous study of^34,35^ that provided extensive information regarding refactoring and source code metrics for 7 open-source Java systems (Table 1). It is composed of 37 releases of these systems and is characterized by a comprehensive manual validation process that guarantees the accuracy and reliability of the data. The RefFinder tool automatically extracted all the refactoring instances to ensure the completeness of the dataset and reduce the risk of human error. The refFinder tool was utilized to identify and categorize types of refactoring. Throughout all the projects and releases a total of 1,820 instances of refactoring were detected by refFinder. These refactorings encompassed a range of operations including extracting methods, reorganizing code, and renaming. The dataset's unique feature is that each refactoring is mapped to source code elements at the method and class levels, including precise version and line information, allowing for the replication of empirical investigations. The dataset used in this research encompasses a range of refactoring types, with 23 classes and 19 methods. The data repository is available from^34,35^ at http://www.inf.u-szeged.hu/~ferenc/papers/RefactDataSet/.

**Table 1:** Refactoring types in the dataset (used in Case 1, Case 2, and Case 3).

Abbreviation	Refactoring type
R1	REMOVE_PARAMETER
R2	ADD_PARAMETER
R3	INTRODUCE_EXPLAINING_VARIABLE
R4	REMOVE_ASSIGNMENT_TO_PARAMETERS
R5	CONSOLIDATE_COND_EXPRESSION
R6	INTRODUCE_EXPLAINING_VARIABLE
R7	CONSOLIDATE_DUPLICATE_COND_FRAGMENTS
R8	EXTRACT_METHOD

To prepare the data to be used for the RcRV visualization tool, the dataset preprocessing phase was conducted. This phase can be summarized as follows:

1. Deleting extra data that is unnecessary for our approach (e.g., metric data).
2. Splitting the data into two files, one for the classes and one for the methods that have refactoring.
3. Deleting every class and method with no refactoring.
4. Linking each method with their respective class.
5. Deleting all refactoring types that are not associated with any class or method.
6. Creating files with the necessary data for each case.

### Implementation

To develop our tool for refactoring, we used Python to generate the radar charts used for visualization. For refactoring purposes, we explored four cases:

- Case 1: Visualization of multiple refactoring for multiple methods in one specific class with a refactoring counter.
- Case 2: Visualization of one refactoring type for multiple methods in multiple classes with a refactoring counter.
- Case 3: Visualization of multiple refactoring for multiple classes with a refactoring counter.
- Case 4: Visualization of multiple refactoring over evolution with a refactoring counter.

**Figure 2:**
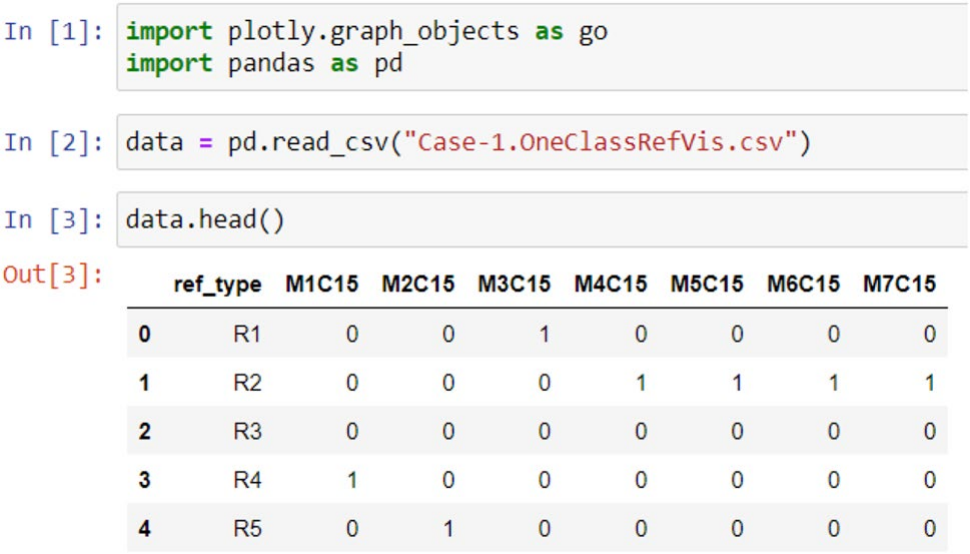
Code snippet for case 1.

**Figure 3:**
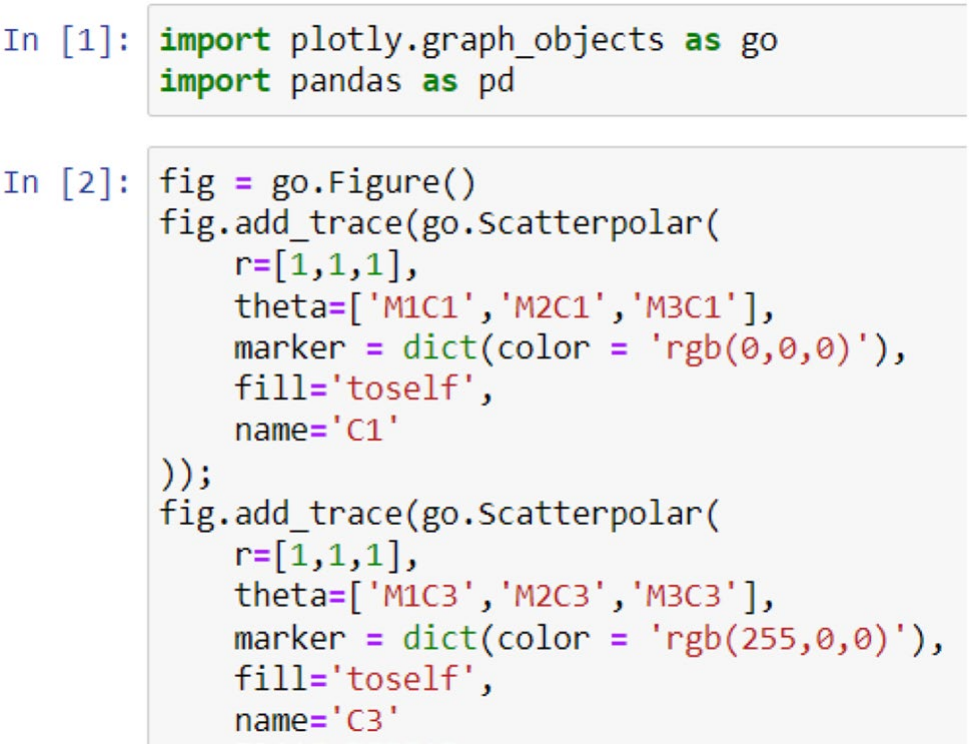
Code snippet for case 2.

**Figure 4:**
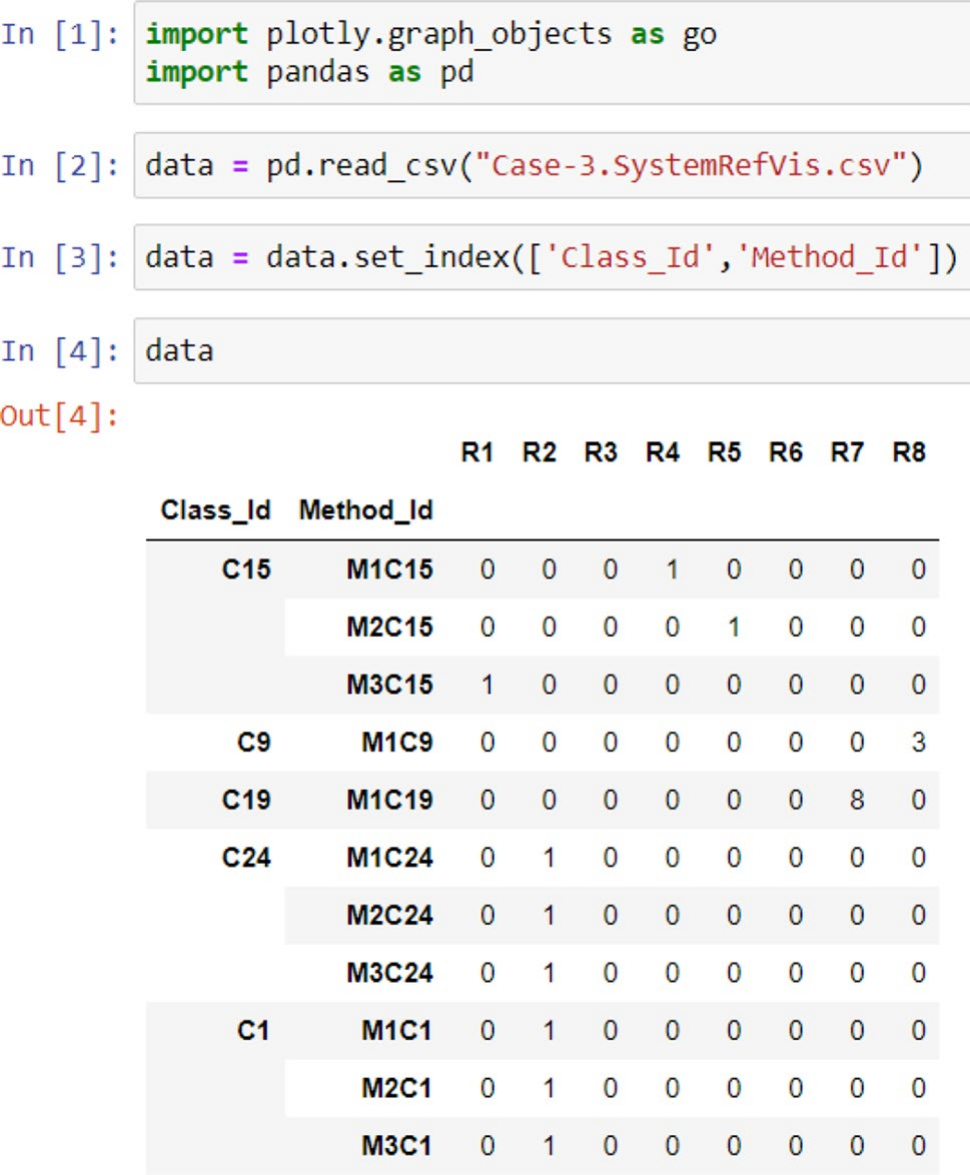
Code snippet for case 3.

**Figure 5:**
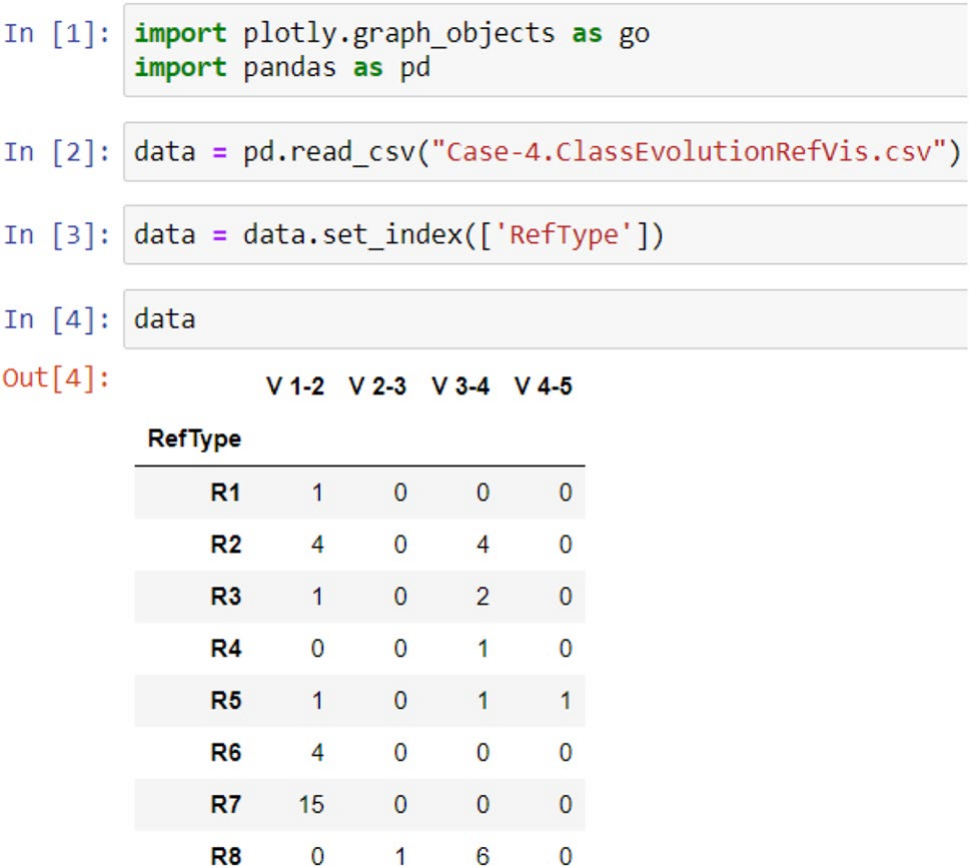
Code snippet for case 4 [refactoring types and their frequency in the dataset over evolutionary data from version 1 to version 5].

As illustrated in Figs. 2, 3, 4, and 5, the used libraries are plotly for visualization, pandas for data manipulation and analysis. These figures illustrate the code developed for generating the four cases: Case 1, Case 2, Case 3, and Case 4, respectively.

There are several types of refactoring in the dataset. The types of refactoring used in Case 1, Case 2, and Case 3 are shown in Table 1. The types of refactoring used in Case 4 are shown in Table 2.

**Table 2:** Refactoring types in the dataset (used in Case 4).

Abbreviation	Refactoring type
R1	REMOVE_PARAMETER
R2	ADD_PARAMETER
R3	INTRODUCE_EXPLAINING_VARIABLE
R4	CONSOLIDATE_COND_EXPRESSION
R5	CONSOLIDATE_DUPLICATE_COND_FRAGMENTS
R6	EXTRACT_METHOD
R7	RENAME_METHOD
R8	INTRODUCE_NULL_OBJECT

### Replication

To replicate the refactoring visualization tool, the code is accessed at https://github.com/AhmedKitt/VisualizeRefactoring.

All the output figures of Cases 1–4 are able to be dealt with in an active way in this link: https://ahmedkitt.github.io/VisualizeRefactoring/figures.html.

## RcRV approach and visualization results

In this study, we propose a new approach called Radar Chart Refactoring Visualization (RcRV) to visualize the refactoring of source code across multiple software releases. RcRV represents refactoring events as rings, allowing the viewer to track refactoring trends over time for various refactoring types, methods, and classes. The resulting graphs offer a concise and informative representation of the refactoring evolution in the software system, facilitating analysis of historical trends and prediction of future directions. Identifying potential refactoring candidates before further system evolution is a crucial benefit of this visualization approach. The analysis of past behavior includes four possible cases:

- Case 1: Visualization of multiple refactoring for multiple methods in one specific class with a refactoring counter.
- Case 2: Visualization of one refactoring type for multiple methods in multiple classes with a refactoring counter.
- Case 3: Visualization of multiple refactoring for multiple classes with a refactoring counter.
- Case 4: Visualization of multiple refactoring over evolution with a refactoring counter.

### Case 1: visualization of multiple refactoring for multiple methods in one specific class with a refactoring counter.

In this case, the radar chart has multiple circles that represent the occurrence of refactoring in one specific class (e.g., C15). For that class, there are multiple methods that may get refactored (e.g., M1C15, M2C15, and M3C15 are methods 1, 2, and 3 in class 15). The methods are denoted by different colors. The radius is numbered (0, 1, 2, 3, …, m) depending on the frequency of a specific refactoring type that occurred in that specific class. There are multiple types of refactoring (R1, R2, R3, …., Rn) distributed over the outer circle. There might be different colored radial lines with different lengths depending on the frequency of refactoring that occurred for each method. If no colored radial line exists for a specific refactoring type, this means that refactoring did not occur.

**Figure 6:**
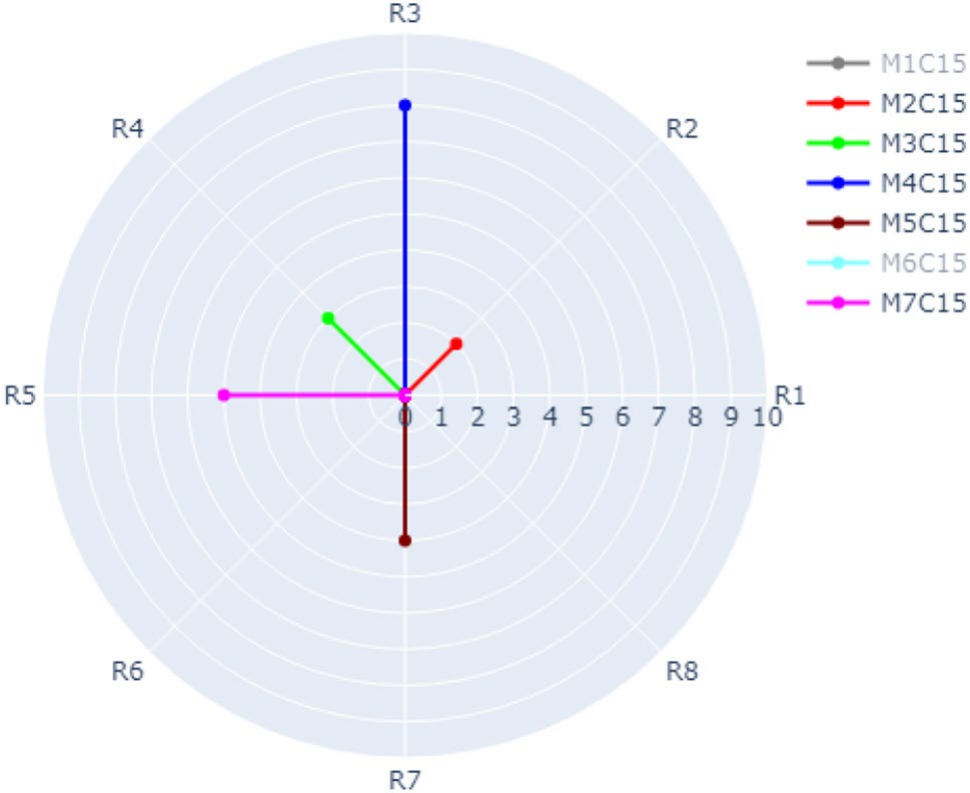
Visualization of Case 1 with M2, M3, M4, M5, and M7 of Class C15 switched on.

**Figure 7:**
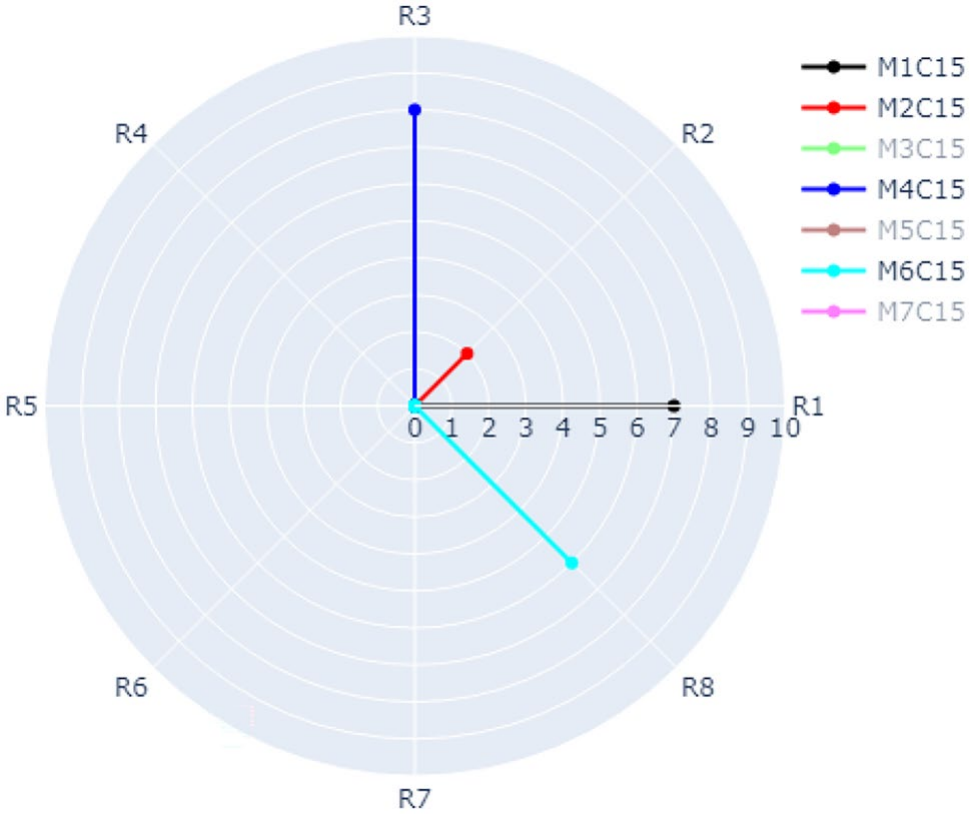
Visualization of Case 1 with M1, M2, M4, and M6 of Class C15 switched on.

**Figure 8:**
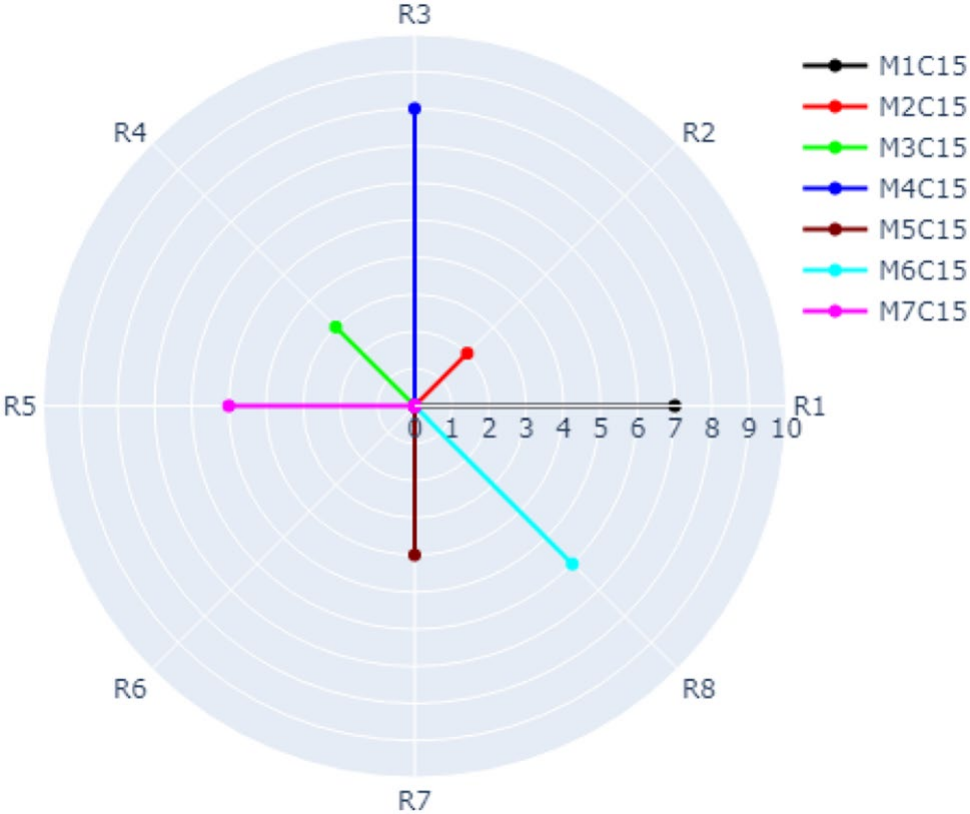
Visualization of Case 1 with all methods of Class C15 switched on.

The visualization of Case 1 is depicted in Fig. 6. According to our dataset, there are eight refactoring types distributed over the outer circle (R1, R2, …., R8) as shown in Fig. 6. There are seven methods (M1, M2, …, M7) in Class 15 represented by different colors. For example, the frequency of R3 (INTRODUCE_EXPLAINING_VARIABLE) in Method 4, Class 15 is 8 times represented by the blue radial line. The frequency of R2 (ADD_PARAMETER) in Method 2, Class 15 is 2 times (represented by the red line). The frequency of R4 (REMOVE_ASSIGNMENT_TO_PARAMETERS) in Method 3, Class 15 is 3 times (represented by the green line). It is possible in our approach to switch on and off the methods you want to show in the radar chart. Figure 6 shows only M2, M3, M4, M5, M8. Figure 7 shows M1, M2, M4, M6. Figure 8 shows the default case with all methods switched on.

### Case 2: visualization of one refactoring type for multiple methods in multiple classes with a refactoring counter

For case 2, the radar chart has multiple circles that represent the occurrence of one specific refactoring type (e.g., R1) for multiple methods and several classes distributed over the outer circle. There are nine classes (C1, C3, C5, C10, C14, C15, C16, C23, C24) represented by different colors. For example, M1C5 represents method 1 in Class 5). For each class, there are multiple methods that may get refactored (e.g., M1C1, M2C1, and M3C1 are methods 1, 2, and 3 in class 1). For the specific refactoring (i.e., R1), the radius is numbered (0, 1, 2, 3, …, n) depending on the frequency of refactoring that occurred for R1. The methods that get refactored in one single class are grouped together depending on the frequency of refactoring that occurred for each method.

**Figure 9:**
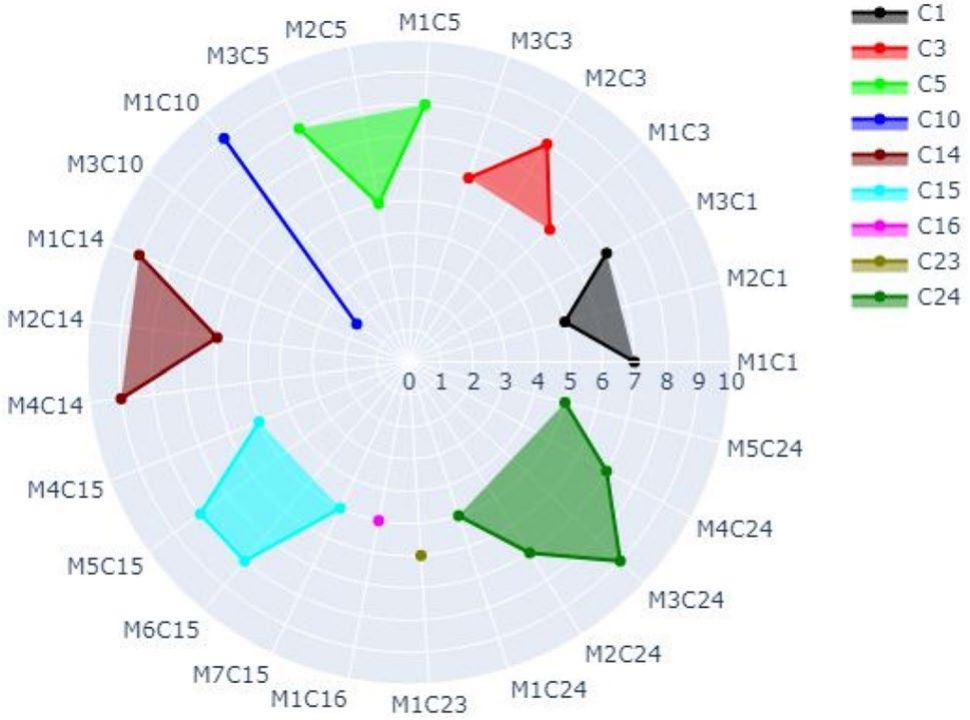
Visualization of Case 2 for all methods in all Classes that have refactoring R1 and their frequency.

As an example, Fig. 9 shows the refactoring type R2 (ADD_PARAMETER).For Class 1 (represented by black color), the frequency of M1C1, M2C1, and M3C1 is 7, 5, and 7, respectively. For Class 24 (represented by olive green), there are five methods that get refactored. That is, M1C24, M2C24, M3C24, M4C24, and M5C24. The refactoring frequency of R1 in these methods is 5, 7, 9, 7, and 5, respectively.

In other examples, M1C16 got refactored 5 times (denoted by the purple dot). M1C23 shows that Method 1 in Class 23 (the brown dot) was refactored 6 times. The blue radial line shows that the two methods M1 and M3 of Class 10 got refactored 9 and 2 times, respectively.

As previously mentioned, you can switch on and off the classes to appear in the radar chart by clicking on the class or multiple classes.

### Case 3: visualization of multiple refactoring for multiple classes with a refactoring counter

In this case, the radar chart has multiple circles that represent the occurrence of multiple refactoring types (e.g., R1, R2, R3, …., Rm) in several classes (e.g., C1, C2, C15). The refactoring types are distributed over the outer circle. The classes are denoted by different colors. The radius is numbered (0, 1, 2, 3, …, n) depending on the frequency of a specific refactoring that occurred in these classes. There might be different colored radial lines with different lengths depending on the frequency of refactoring that occurred for each class.

**Figure 10:**
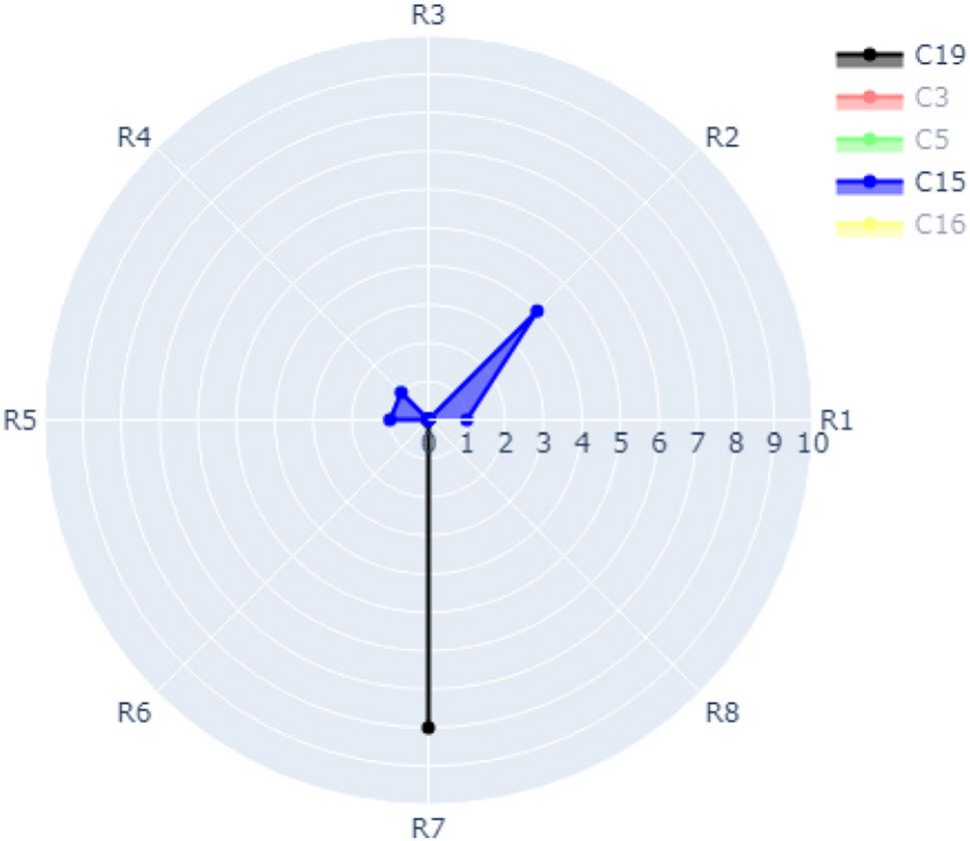
Visualization of Case 3 with Classes C15 and C19 switched on.

As shown in Fig. 10, for Class C15 the refactoring type R1 (REMOVE_PARAMETER), R4 (REMOVE_ASSIGNMENT_TO_PARAMETERS), and R5 (CONSOLIDATE_COND_EXPRESSION) were refactored once. R2 (i.e., ADD_PARAMETER) was refactored four times. For Class C19, the refactoring type R7 (CONSOLIDATE_DUPLICATE_COND_FRAGMENTS) was refactored 8 times.

### Case 4: visualization of multiple refactoring over evolution with a refactoring counter

In case 4, the radar chart has multiple rings that represent the occurrence of multiple refactoring types (e.g., R1, R2, R3, …., Rm) over evolution. The refactoring types are distributed over the outer circle. The evolution of versions is denoted by different colours (e.g., V 1–2 represents the evolution from version 1 to version 2). The radius is numbered (0, 1, 2, 3, …, n) depending on the frequency of a specific refactoring that occurred for a specific version. There might be different coloured radial lines with different lengths depending on the frequency of refactoring that occurred for each evolution.

**Figure 11:**
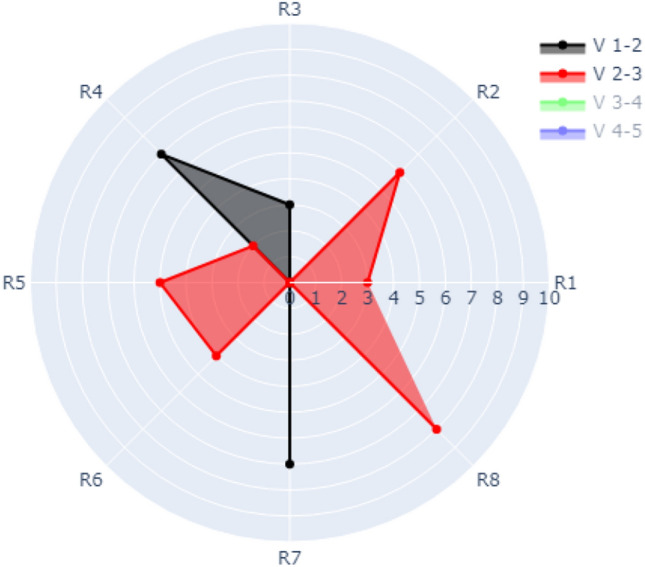
Visualization of Case 4 with all refactoring of V 1–2 and V 2–3 switched on.

Figure 11 presents visualization of data over evolution for Version1 to Version 2, and Version 2 to Version 3, (Version 3–4 and Version 4–5 are switched off). The figure shows that the following refactoring occurred within V 1–2 (black colour) which are R3, R4, and R7. The frequency of R3, R4, and R7 occurred3, 7 and 7 times, respectively. The visualization of data over evolution for Version 2 to Version 3 (red colour) shows that the frequency of the refactoring types R1, R2, R4, R5, R6, and R8 are 3, 6, 2, 5, 4, and 8, respectively.

**Figure 12:**
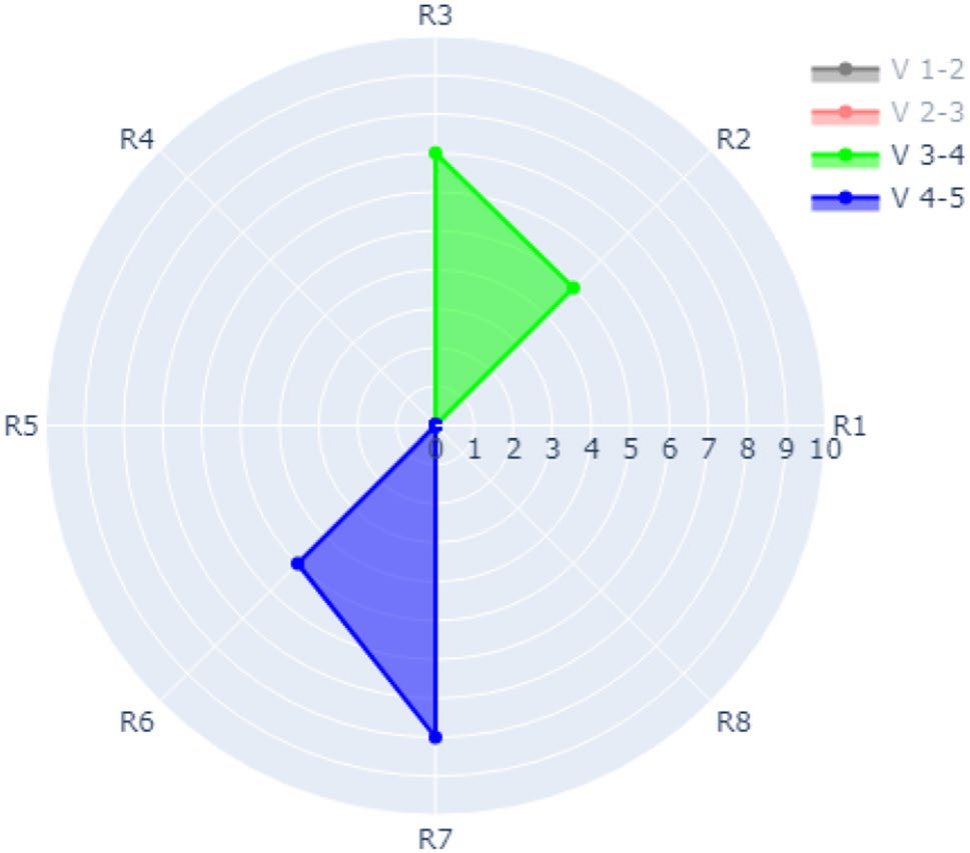
Visualization of Case 4 with all refactoring of V 3–4 and V 4–5 switched on.

The refactoring visualization for Version 3–4 and Version 4–5 is shown in Fig. 12 (Version 1–2 and Version 2–3 are switched off). As shown in the figure, the refactoring of V 3–4 for R2 occurred 5 times and 7 times for R3. The refactoring frequency of V4-5 for R6 is 5 times while it is 8 times for R7.

**Figure 13:**
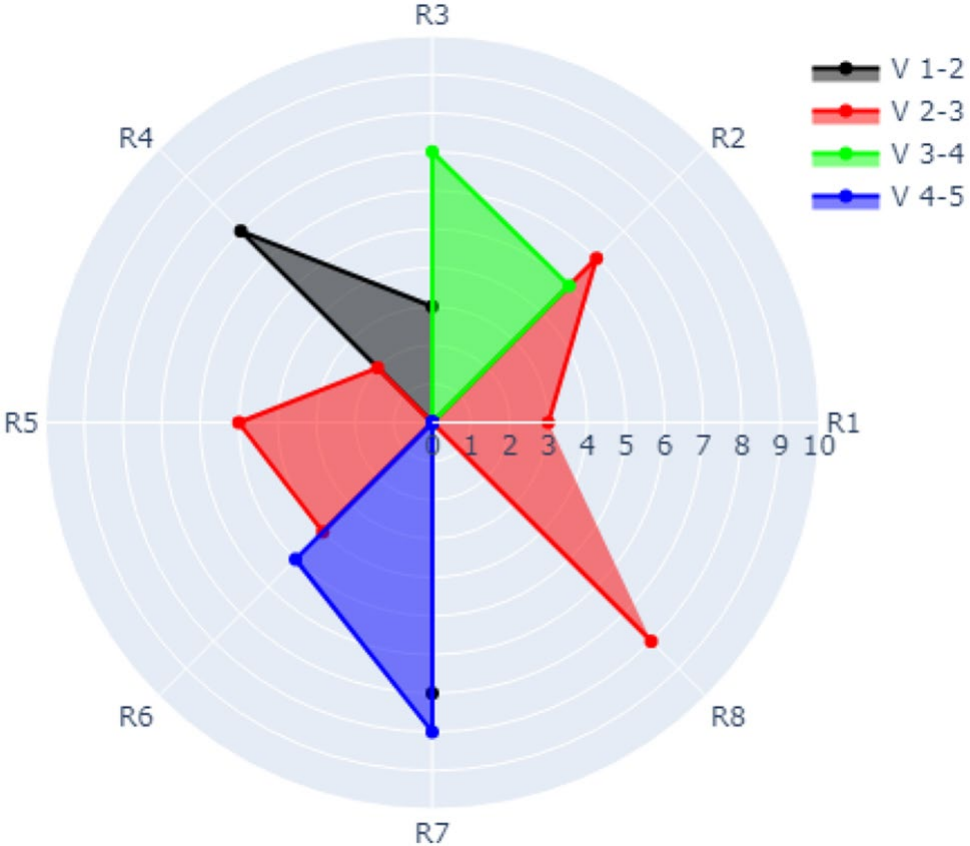
Visualization of Case 4 with all evolutionary data from V 1–2 to V 4–5 switched on.

Another example of Case 4 is shown in Fig. 13 where evolution over data for all versions is switched on for all version which combines Figs. 11 and 12.

## Evaluation of the refactoring visualization tool

To evaluate the usability and usefulness of the developed tool, we adopted a controlled experiment research design to assess the impact of the visualization tool on software refactoring activities^36^. The evaluation study encompassed multiple phases, beginning with the organization of focused group sessions. These focus group sessions aimed at familiarizing the participating developers with the visualization tool and providing an understanding of its functionalities. Following this preliminary stage, a cohort of 80 software developers was recruited to take part in the subsequent evaluation of the visualization tool. The participants in our evaluative study comprised software developers employed by a prominent private-sector organization based in Jordan. Participant selection was based on their relevance to the evaluation study's objectives and willingness to participate. Table 3 summarizes the demographic distribution of participants.

**Table 3:** Demographic characteristics of participants.

Demographic characteristics	Number of participants	Percentage
Age group		
25–34 years	45	56.25
35–44 years	25	31.25
45 years and above	10	12.50
Gender		
Male	55	68.75
Female	25	31.25
Coding experience		
Beginner	15	18.75
Intermediate	35	43.75
Advanced	30	37.50
Frequency of refactoring		
Occasionally	15	18.75
Regularly	40	50.00
Frequently	25	31.25

The participants were allocated into two distinct groups: Group 1 and Group 2. Each group consisted of 40 developers. Group 1 was assigned the role of the control group, wherein participants did not have access to the visualization tool during the refactoring tasks. Group 2 constituted the experimental group and was provided access to the visualization tool for the same tasks and operated under equivalent conditions. Both groups were subjected to a set of predefined refactoring tasks. These tasks were selected to encompass a variety of refactoring activities commonly encountered in software development, ensuring the experimental setup reflected real-world scenarios. The refactoring tasks were executed within the context of an open-source project, thus establishing a realistic environment for the experiment.

Quantitative metrics were employed to assess the impact of the visualization tool. These metrics included cyclomatic complexity; lines of code; code duplication percentage; maintainability index; depth of inheritance; class coupling; method complexity; and test coverage^37,38^. Cyclomatic complexity was used to gauge the complexity of control flow in the refactored code. Code duplication percentage identified instances of duplicated code. The maintainability index quantified the maintainability of the refactored code, while class coupling measured dependencies between classes. Test coverage was used to evaluate the extent of code coverage achieved by automated tests^2^. Table 4 shows the code quality metrics employed in our study which are among the most common and widely used metrics in software engineering. The inclusion of these metrics in our evaluation study aims to provide a quantitative assessment of how the RcRV influences various aspects of code quality, maintainability, and development efficiency. Therefore, allowing us to calculate the percentage of improvement for each metric between the two groups and provide data-driven conclusions about the tool’s benefit and the effectiveness of our visualization tool in the refactoring process.

**Table 4:** The results of the comparison conducted between Group 1 and Group 2.

Metric	Group 1 without visualization tool	Group 2 with visualization tool	Improvement
Cyclomatic complexity	18	10	Lower is better
Lines of code	700	500	Lower is better
Code duplication	15%	8%	Lower is better
Maintainability index	65	80	Higher is better
Depth of inheritance	5	3	Lower is better
Class coupling	50	40	Lower is better
Method complexity	8	6	Lower is better
Test coverage	75%	85%	Higher is better
No. of refactoring	6	16	Higher is better
Time	55 s	22 s	Lower is better

Upon completion of the refactoring tasks, the gathered data was subjected to thorough analysis. A comparative assessment of the metrics between Group 1 and Group 2 was conducted to identify any discernible differences resulting from the utilization of the visualization tool. The analysis focused on analyzing the aforementioned code metrics, the number of refactoring performed, and the time needed to apply the refactoring tasks. Additionally, the p-value was calculated for the two groups, and it was less than 0.05 which implies that the two groups are statistically different^39^.

Table 4 outlines the results obtained from the comparison between Group 1 (without the visualization tool) and Group 2 (with the visualization tool) regarding various key metrics. These metrics provide insights into the impact of utilizing the visualization tool during refactoring activities as follows:

*Cyclomatic Complexity:* Group 2 exhibited a lower cyclomatic complexity value (10) compared to Group 1 (18), indicating that the visualization tool contributed to reducing the complexity of the control flow in the codebase.

*Lines of Code:* Group 2 showed lower lines of code count (500) as opposed to Group 1 (700), suggesting that the visualization tool aided in streamlining and optimizing code length.

*Code Duplication:* Group 2 demonstrated a lower code duplication percentage (8%) in contrast to Group 1 (15%), indicating that the visualization tool effectively assisted in mitigating duplicated code segments.

*Maintainability Index:* The maintainability index was higher for Group 2 (80) than for Group 1 (65), highlighting that the visualization tool positively influenced the overall maintainability of the codebase.

*Depth of Inheritance:* Group 2 exhibited a lower depth of inheritance value (3) compared to Group 1 (5), suggesting that the visualization tool contributed to a more concise and comprehensible class hierarchy.

*Class Coupling:* The class coupling value was lower for Group 2 (40) as opposed to Group 1 (50), indicating that the visualization tool facilitated the reduction of interdependencies between classes.

*Method Complexity:* Group 2 showed a lower method complexity value (6) in comparison to Group 1 (8), suggesting that the visualization tool aided in simplifying individual method logic.

*Test Coverage:* Group 2 achieved a higher test coverage percentage (85%) compared to Group 1 (75%), indicating that the visualization tool positively influenced the comprehensiveness of automated testing.

*Number of Refactoring:* Group 2 performed a higher number of refactoring tasks (16) in contrast to Group 1 (6), signifying that the visualization tool potentially enabled more frequent and effective refactoring.

*Time:* Group 2 exhibited a lower average time per refactoring task (22 s) compared to Group 1 (55 s), suggesting that the visualization tool led to faster and more efficient refactoring activities.

The collective results indicate that the utilization of the visualization tool had a positive influence on various aspects of refactoring. Group 2 demonstrated improvements in code quality, maintainability, complexity, test coverage, and efficiency, as evidenced by the metrics presented in Table 4.

**Figure 14:**
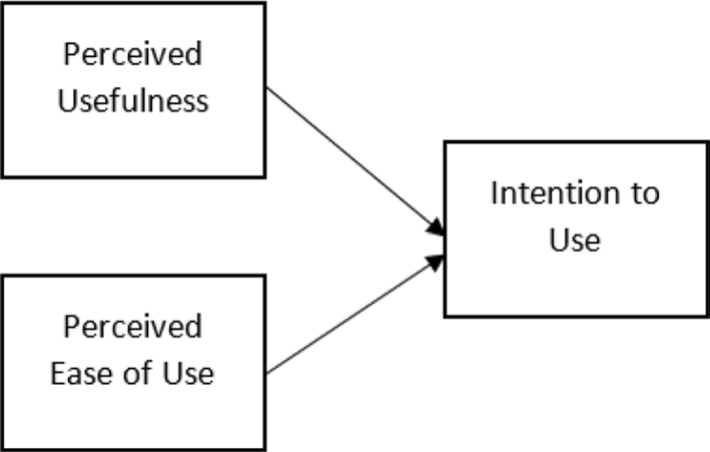
Technology acceptance model^40^.

The next step in the evaluation part was the distribution of a questionnaire for Group 2 that used our visualization refactoring tool. The questionnaire was designed based on the constructs of the “Technology Acceptance Model” developed by Davis et al.^40^ (Fig. 14), and the questionnaire items proposed by^41^. Appendix 1 shows the scale items adopted to measure the usefulness, ease of use, and intention to use.

To evaluate these constructs, the following metrics were retrieved:

1. Cronbach's alpha (α) to measure the internal consistency; cut off ≥ 0.70.
2. Composite Reliability (CR) to measure the reliability; cut off ≥ 0.70.
3. Average Variance Extracted (AVE) to measure the validity; cut off ≥ 0.50.

Table 5 shows that the values retrieved for internal reliability and validity have met the minimum requirements as suggested in the literature^42^.

**Table 5:** The results of internal consistency (α), composite reliability (CR), and validity (AVE).

Constructs	α	CR	AVE
Usefulness	0.830	0.880	0.690
Ease of use	0.850	0.890	0.630
Intention to use	0.800	0.850	0.700

As shown in Table 5, Group 2 perceives that the RcRV is useful, easy to use, and they intend to use it in the future. According to the obtained results, we can conclude that our proposed visualization tool for refactoring is considered useful to developers, appealing, and easy to use.

## Implications

The implications of this research are multifaced, highlighting the benefits of the RcRV which can assist professionals, educators, researchers, and developers in the field of software engineering as follows.

Practitioners:

1. The newly introduced visualization tool stands as a valuable tool that can assist professionals, like software developers and software engineers in dealing with the complexity of existing refactoring tools. It offers representations that simplify the comprehension of complex code modifications.
2. This innovation empowers developers to explore the evolution of refactoring within software systems. By enabling the analysis of historical trends of refactoring and the prediction of future directions, developers can identify potential areas for refactoring. This fosters a proactive approach to code enhancement and maintenance, enhancing the overall software quality.
3. The RcRV tool has the potential to improve refactoring tasks by offering insights into how different changes can affect the overall structure and maintainability of the software.
4. The visualization of refactoring actions promotes the adoption of best practices in the software engineering field which in turn ensures code quality and readability.
5. Tool builders in the software development industry may integrate or expand the RcRV tool within their software development environments. This integration will improve refactoring capabilities.

Educators:

1. Educators can utilize our RcRV tool as a teaching aid in the software engineering discipline. It provides interactive demonstrations of the refactoring process, which helps students to better understand abstract concepts.
2. Utilizing the RcRV tool can aid students in gaining hands-on experience with refactoring techniques in a controlled setting utilizing a user-friendly tool that can improve their skills and comprehension.
3. Educators can make use of our RcRV tool to illustrate the benefits and rationale behind implementing refactoring strategies.

Researchers:

1. Researchers could use the RcRV tool to gather data on the processes and practices of refactoring. The tool is capable of capturing user interactions and offering data that can be used in empirical studies.
2. Further experiments using this tool may enable researchers to assess the efficiency of visualizing refactoring or discover novel methods for visualizing refactoring tasks.
3. The visualizations provided by the RcRV tool can effectively assist researchers in analyzing and comprehending refactoring data.

The empirical evaluation of the proposed tool suggests its practicality, user-friendliness, and ease of use to developers, highlighting its crucial role in improving the software development process.

## Conclusion

Software refactoring is an essential practice for software engineers to improve the quality of their codebase. Visualizations are essential tools for understanding the impact of refactoring operations. Existing visualization techniques have several limitations that make them less effective for visualizing the effects of software refactoring. In this paper, we propose a novel approach to visualize software refactoring using radar charts. Our proposed approach can help developers highlight areas of refactoring for multiple refactoring, multiple methods, and multiple classes over evolution, understand the impact of code changes, and make informed decisions during the refactoring process. In conclusion, this paper highlights the importance of refactoring visualization in software engineering and the lack of effective visualizations to aid developers in understanding the impact of these changes. The use of radar charts provides developers with a clear and concise view of the changes made during refactoring, allowing them to identify areas of improvement in the code's quality. This visualization approach can save time and effort during the development process by allowing developers to quickly identify potential issues in the code that violate design principles and are susceptible to bugs. As such, this paper offers a valuable contribution to the field of software engineering, providing developers with a powerful tool to improve code quality and streamline the development process.

## Limitations and future work

The application of radar charts as a means of evaluating the impact of refactoring may not be universally applicable to all software projects, particularly those with a smaller codebase or fewer dimensions of the code that require improvement. It's important to note that while our RcRV approach offers a useful visualization tool for tracking software refactoring that is valid for large and small datasets and produces visualization for different levels (e.g., methods, classes, one refactoring type, n-releases), its applicability may be limited to certain types of software projects. Smaller codebases or projects with fewer dimensions that require improvement may not benefit as much from this approach. Different projects may require diverse approaches depending on their needs and objectives. Further, as our work was initially based on Ref-Finder due to the available dataset and the constraints of our research timeline, we recognize the limitation in the choice of tool. In future iterations of our work, we plan to explore the use of more accurate tools such as Refactoring Miner to enhance the accuracy of our refactoring data. Further, we value the insights gained from our RcRV evaluation study. Nevertheless, it's important to acknowledge certain limitations, particularly the small sample size, which may impact its broader applicability. Additionally, we encountered challenges in accessing a larger participant pool due to resource constraints. Future research can address these limitations by expanding the sample size and combining qualitative methods to gain deeper insights into developers' experiences.

The proposed RcRV approach offers a promising tool for software developers to visualize and track the evolution of refactoring in their codebases. Further investigation is necessary to evaluate our visualization tool in real-world projects. The use of radar charts as proposed may be extended to include additional dimensions of the code, such as security or performance, to provide a more comprehensive view of software quality. Empirical studies involving software developers working on real-world projects could be conducted to evaluate the effectiveness of the approach. Additionally, the approach could be integrated with existing refactoring tools to offer developers a more streamlined and intuitive approach to visualizing the impact of their code alterations. These extensions may help identify potential areas for improvement in future releases, making the RcRV approach a valuable tool for improving software quality.

## Data Availability

The data presented in this study are available and can be accessed at (https://github.com/AhmedKitt/VisualizeRefactoring).

## Appendix

See Table 6.

**Table 6:** The scale items adopted to measure the usefulness, ease of use, and intention to use RcRV.

Construct	Item	SA	A	N	D	SD
Usefulness	UF1: using RcRV in my job would enable me to accomplish tasks more quickly (quick)					
	UF2: using RcRV would improve my job performance (job performance)					
	UF3: using RcRV in my job would increase my productivity (increase productivity)					
	UF4: using RcRV would enhance my effectiveness on the job (effectiveness)					
	UF5: using RcRV would make it easier to do my job (makes job easier)					
	UF6: I find RcRV useful in my job (useful)					
Ease of use	EU1: learning to operate RcRV is easy for me (easy to learn)					
	EU2: I find it easy to get the RcRV to do what I want it to do (clear and understandable)					
	EU3: my interaction with RcRV would be clear and understandable (controllable)					
	EU4: It was easy to become skillful using RcRV (Skillful)					
	EU5: It is easy to remember how to perform tasks using RcRV (Remember)					
	EU6: I find RcRV easy to use (Easy to use)					
Intention to use	IU1: assuming RcRV would be available on my job, I predict that I will use it on a regular basis in the future					
	IU2: I would prefer to perform refactoring tasks using RcRV					
	IU3: I intend to use RcRV in the future					

*SA* strongly agree, *A* agree, *N* neutral, *D* disagree, *SD* strongly disagree.
